# Electroacupuncture for the Prevention of Perioperative Neurocognitive Disorder in Older Patients Undergoing General Anesthesia: Protocol for a Systematic Review and Meta-Analysis

**DOI:** 10.2196/84010

**Published:** 2025-12-30

**Authors:** Changle Wu, Xuqiang Wei, Ke Wang, Jia Zhou

**Affiliations:** 1 Acupuncture Anesthesia Clinical Research Institute, Yueyang Hospital of Integrated Traditional Chinese and Western Medicine, Shanghai University of Traditional Chinese Medicine, No. 110 Ganhe Road, Shanghai, 200437, China, 86 2120256344; 2 Shanghai Acupuncture Clinical Research Center, Yueyang Hospital of Integrated Traditional Chinese and Western Medicine, Shanghai University of Traditional Chinese Medicine, Shanghai, China

**Keywords:** electroacupuncture, perioperative neurocognitive disorder, postoperative cognitive dysfunction, postoperative delirium, older adults, meta-analysis, Preferred Reporting Items for Systematic Reviews and Meta-Analyses, PRISMA

## Abstract

**Background:**

Perioperative neurocognitive disorder (PND) is a prevalent complication among older patients undergoing general anesthesia, imposing significant burdens on individuals, health care systems, and society. While electroacupuncture shows promise for PND prevention, current evidence remains inconclusive.

**Objective:**

This study aims to critically evaluate the effectiveness and safety of perioperative electroacupuncture for PND prevention in older patients undergoing surgery under general anesthesia.

**Methods:**

A comprehensive literature search will be conducted in 8 electronic databases (PubMed, Embase, Web of Science, Cochrane Library, China National Knowledge Infrastructure, Chongqing VIP Chinese Science and Technology Periodical Database, Wan Fang Database, and China Biomedical Literature Database) and 3 clinical trial registries from inception to March 16, 2025. The search strategy aims to identify all relevant randomized controlled trials evaluating perioperative electroacupuncture for PND prevention in older patients (aged ≥60 years) undergoing general anesthesia. The primary outcome will be the incidence of PND. Secondary outcomes will include (1) neuropsychological assessment scores (Mini-Mental State Examination and Montreal Cognitive Assessment), (2) serum inflammatory biomarker levels (interleukin-1β, interleukin-6, and tumor necrosis factor-α), (3) serum neurological damage marker levels (neuron-specific enolase and S100 calcium-binding protein β), and (4) safety outcomes (incidence of adverse events). Two independent reviewers will perform study selection, data extraction, and methodological quality assessment using the revised Cochrane risk of bias tool for randomized trials. All statistical analyses will be conducted in RevMan 5.4 using suitable meta-analysis models based on heterogeneity testing. The certainty of evidence will be evaluated using Grading of Recommendations Assessment, Development and Evaluation (GRADE).

**Results:**

The study selection process will be presented through a PRISMA (Preferred Reporting Items for Systematic Reviews and Meta-Analyses) flow diagram, detailing the number of records identified, screened, and included. Characteristics of eligible studies will be summarized in evidence tables, including study designs and populations, intervention protocols, and outcome measures. The results will be visualized through a risk of bias graph, forest plots displaying pooled effect estimates with 95% CIs, and funnel plots for publication bias evaluation (when ≥10 studies are available). This protocol is currently in the active phase. The literature search has been completed as of April 2025, with an updated search planned until December 31, 2025. Data extraction is scheduled to commence on January 15, 2026, followed by data analysis starting February 1, 2026. Results are expected to be submitted for publication in March 2026.

**Conclusions:**

The effectiveness and safety of perioperative electroacupuncture for PND prevention in older patients undergoing general anesthesia remain uncertain. This systematic review will provide an evidence-based evaluation of perioperative electroacupuncture’s effectiveness in preventing PND, offer practical recommendations for optimizing surgical care for older adults, and identify knowledge gaps to inform future research.

## Introduction

Perioperative neurocognitive disorder (PND), the revised diagnostic terminology replacing postoperative cognitive dysfunction (POCD), is a common surgical complication characterized by impairment and decline across multiple cognitive domains, including perception, executive function, memory, orientation, language, and attention [1]. According to the 2018 International Nomenclature Consensus [2], PND encompasses 3 components: preexisting cognitive impairment, POCD, and postoperative delirium (POD), meaning postoperative PND specifically refers to both POCD and POD. PND arises from an interplay of multiple risk factors, with older patients undergoing major surgeries under deep anesthesia being particularly vulnerable [1,3,4]. Epidemiological data reveal that PND affects 12% to 25.8% of patients older than 60 years undergoing general surgery [1]. However, in those undergoing major surgeries, such as certain orthopedic and noncardiac procedures, the incidence can rise to as high as 50% [1,3], underscoring the profound impact of surgical invasiveness and trauma. The cognitive impairment may persist for extended periods or become permanent [1,3], leading to serious consequences such as prolonged hospitalization, increased medical expenses, frequent readmissions, diminished quality of life, and elevated risks of dementia and mortality [5-8].

Despite dexmedetomidine showing some therapeutic promise, its use remains limited to severely agitated or distressed cases due to unavoidable side effects [1,3], while other pharmacological approaches lack convincing evidence for preventing or slowing PND progression [9]. These limitations have increased the emphasis on nondrug preventive measures [1,3,9,10]. Electroacupuncture, as an alternative therapy, has emerged as a leading candidate for PND due to its safety, convenience, and high efficacy [11], with clinical trials [12-16] demonstrating perioperative electroacupuncture’s effectiveness in reducing PND incidence and improving postoperative cognitive recovery in older patients undergoing major surgeries (knee replacement, total hip arthroplasty, subtotal gastrectomy, and spine procedures) and mechanistic studies [17-21] elucidating its multimodal neuroprotective effects, including neuroinflammation attenuation, synaptic plasticity remodeling, microglial activation suppression, oxidative stress mitigation, and cerebral perfusion enhancement.

Nevertheless, current evidence regarding perioperative electroacupuncture for PND prevention in older adults undergoing surgery under general anesthesia remains inconclusive, primarily owing to significant clinical heterogeneity (eg, age, surgical types, anesthesia methods, and electroacupuncture stimulus parameters), methodological heterogeneity (eg, diagnostic criteria, assessment tools, and outcome assessment time points), potential methodological biases (high risk of performance and detection bias induced by inadequate blinding), and inadequate statistical power (small sample sizes) in existing studies [12-16,22]. Previous systematic reviews and meta-analyses [23-28] have primarily focused on investigating the effects of transcutaneous electrical acupoint stimulation on isolated outcomes (eg, POCD or POD alone), failing to address PND as a unified clinical entity or leverage interconnected pathophysiological insights. Only 2 studies [16,22] have investigated the effects of electroacupuncture on PND. One study [16] specifically observed older patients but included both general anesthesia and spinal-epidural anesthesia; the other [22] enrolled participants aged 18 years and above, failing to meet the criteria for an older adult population.

Given this, we plan to conduct a comprehensive systematic review and meta-analysis of randomized controlled trials (RCTs) investigating perioperative electroacupuncture for PND prevention in older patients (≥60 years) undergoing general anesthesia. This systematic review and meta-analysis is specifically designed to address the abovementioned challenges by rigorously assessing the risk of bias, investigating heterogeneity through prespecified subgroup analyses, and applying standardized outcome definitions, thereby providing a more nuanced and reliable estimate of electroacupuncture’s true effect. The objectives are threefold: (1) to quantify electroacupuncture’s effectiveness in preventing PND and improving cognitive recovery, (2) to quantitatively analyze electroacupuncture’s modulatory effects on serum inflammatory markers (interleukin-1β [IL-1β], interleukin-6 [IL-6], and tumor necrosis factor-α [TNF-α]) and neurological injury biomarkers (neuron-specific enolase [NSE] and S100 calcium-binding protein β [S100β]), and (3) to objectively examine methodological limitations in existing RCTs. We anticipate that our findings will provide a comprehensive evidence base regarding electroacupuncture’s efficacy and safety. This evidence is poised to inform the optimization of postoperative neurocognitive protection strategies and may offer a compelling, nonpharmacological intervention for integration into Enhanced Recovery After Surgery (ERAS) protocols, particularly for the vulnerable older adults. Furthermore, this review will provide crucial guidance for the design of future high-quality RCTs in this field.

## Methods

### Study Registration

Registration has been completed in the PROSPERO database before data extraction (CRD420251035172). The protocol is reported in compliance with PRISMA-P (Preferred Reporting Items for Systematic Reviews and Meta-Analyses Protocols) [29], and the completed PRISMA-P checklist is provided in Checklist 1. The study will be conducted strictly in accordance with the Cochrane Handbook for Systematic Reviews of Interventions [30] and will adhere to the PRISMA (Preferred Reporting Items for Systematic Reviews and Meta-Analyses) 2020 guidelines [31] to ensure transparent and complete reporting.

### Study Design

We intend to incorporate RCTs that meet predefined eligibility criteria to evaluate the effectiveness and safety of electroacupuncture in preventing PND among older patients undergoing general anesthesia.

### Eligibility Criteria

Studies will be selected according to the criteria outlined in the following sections.

#### Type of Studies

We will include all parallel-group RCTs. Non-RCTs (eg, cohort studies, case reports, and reviews) will be excluded, regardless of their sample size.

#### Type of Participants

Participants must be older patients (age ≥60 years) undergoing elective surgery under general anesthesia, with no preoperative cognitive impairment, as confirmed by the Mini-Mental State Examination (MMSE) or Montreal Cognitive Assessment (MoCA). There are no restrictions on gender, type of surgery, or educational level. Patients with preexisting cognitive disorders according to the MMSE or MoCA or those with cognitive decline caused by neurodegenerative diseases such as Alzheimer disease, mild cognitive impairment, vascular dementia, mental illness, cranial brain trauma, stroke, and other psychiatric conditions before surgery will be excluded.

#### Type of Intervention

The intervention must be perioperative electroacupuncture (preoperative, intraoperative, postoperative, or combined multiphase), with no restrictions on acupoint selection, waveform, frequency, stimulus intensity, or duration.

#### Types of Comparisons

The comparators will receive sham electroacupuncture (nonpenetrating or nonacupoint stimulation), no intervention, or standard care. Studies using electroacupuncture in the control group to compare technical parameters (eg, waveform, frequency, or intensity) will be excluded, as these investigations primarily aim to optimize stimulation protocols rather than evaluate clinical efficacy against blank, routine, or sham controls.

#### Types of Outcome Measures

The primary outcome will be the incidence of PND (POCD or POD). POD is explicitly defined as an acute and fluctuating disturbance in attention and awareness occurring within the first 7 days postoperatively or until discharge [2]. Diagnosis is based on either the Confusion Assessment Method or the Nursing Delirium Screening Scale. POCD is defined as an objective decline in cognitive function, assessed by a battery of neuropsychological tests from preoperative baseline to a postoperative time point (1-7 days, 14 days, 1 month, and 3 months) [2]. A postoperative decline in the MMSE score of ≥1 SD from the preoperative baseline [2] or an absolute postoperative MMSE score of <27 [32] is defined as POCD. Secondary outcomes will include cognitive function scores (assessed by MMSE or MoCA), serum inflammatory biomarker levels (IL-1β, IL-6, and TNF-α), serum neurological damage marker levels (NSE and S100β), and the incidence of adverse events. Studies demonstrating clearly erroneous outcomes or ambiguous diagnostic criteria will be excluded.

#### Other Criteria

Eligible studies may be from any region but must be published in English or Chinese. Studies without preoperative cognitive assessment will be excluded to ensure baseline comparability of cognitive function.

### Information Sources and Search Strategy

A comprehensive search will be conducted in 8 electronic databases, including PubMed, Embase, Cochrane Central Register of Controlled Trials, Web of Science, China National Knowledge Infrastructure, Chongqing VIP Chinese Science and Technology Periodical Database, Wan Fang, and China Biomedical Literature Database, from inception to March 16, 2025. The literature search will be updated to include all studies available up to just before the manuscript’s final submission to capture the most recent evidence. To maximize the scope of review, the ClinicalTrials.gov, Chinese Clinical Trial Registry, International Traditional Medicine Clinical Trial Registry, and gray literature (eg, dissertations) will be additionally searched to identify eligible trials. Furthermore, the reference lists of included studies will be manually screened to supplement relevant literature.

The search strategy will integrate both Medical Subject Headings (MeSH) and free-text terms across four critical domains: (1) population—“aged,” “elderly,” “geriatric,” “perioperative neurocognitive disorder,” “postoperative cognitive dysfunction,” “delirium,” and “cognitive decline”; (2) intervention—“electroacupuncture,” “acupuncture,” and “EA”; (3) context—“general anesthesia,” “surgery,” and “postoperative period”; and (4) study design—“randomized controlled trial” and “RCT.” Boolean operators (AND, OR, NOT) and truncation symbols (*) will be used to ensure optimal balance between search sensitivity and specificity. The detailed search strategy for PubMed is provided in Table 1. The complete, reproducible search strategies for all other databases and clinical trial registries are provided in Multimedia Appendix 1, ensuring full reproducibility as per PRISMA-P guidelines.

**Table 1.** The search strategy for PubMed.

Order	Strategy
#1	(cognition) OR (neurocognitive disorders) OR (cognitive dysfunction) OR (cognition disorders) OR (delirium) [MeSH^a^ Terms]
#2	(cognitive disorders) OR (cognitive impairment) OR (cognitive decline) OR (cognition dysfunction) OR (cognition impairment) OR (cognition decline) OR (cognit*^b^) [Title/Abstract]
#3	#1 OR #2
#4	(postoperative period) OR (anesthesia recovery period) OR (postop*) OR (postoperative*) OR (postoperative) OR (postsurgical*) OR (post anesthesia) OR (after surgery) OR (post-surgery) [Title/Abstract]
#5	#3 AND #4
#6	(postoperative cognitive complications) [MeSH Terms]
#7	(delayed neurocognitive recovery) OR (perioperative neurocognitive disorder) OR (perioperative cognitive disorder) OR (POCD^c^) OR (POD^d^) OR (PND^e^) [Title/Abstract]
#8	#5 OR #6 OR #7
#9	(acupuncture) OR (electroacupuncture) OR (acupuncture therapy) [MeSH Terms]
#10	(aged) [MeSH Terms]
#11	(elderly) OR (older) OR (geriatric) [Title/Abstract]
#12	#10 OR #11
#13	(randomized controlled trial) OR (controlled clinical trial) OR (random*) OR (randomization) OR (randomly) [Title/Abstract]
#14	#8 AND #9 AND #12 AND #13

a MeSH: Medical Subject Headings.

b Asterisks denote the special character that represents zero or more characters in wildcard searching.

c POCD: postoperative cognitive dysfunction.

d POD: postoperative delirium.

e PND: perioperative neurocognitive disorder.

### Study Selection

All identified records will be imported into EndNote (version X20; Clarivate Analytics) for reference management and deduplication. Two independent reviewers (CW and XW) will perform screening based on title, abstract, and full-text assessment. Discrepancies will be discussed and adjudicated by a third reviewer (KW).

### Data Extraction

Two independent researchers (CW and XW) will perform data extraction using a standardized template developed in accordance with the Cochrane Handbook [33], with disagreements resolved by KW. Researchers will extract (1) study characteristics (first author, publication year, nationality, etc), (2) participant demographics (age, sex ratio, sample size, surgical type, etc), (3) intervention details (acupoints, stimulation parameters [waveform, frequency, and intensity], electroacupuncture intervention timing [preoperative, intraoperative, and postoperative], sessions, duration, etc), and (4) outcomes (evaluation indicators [POCD and POD], assessment time points, measurement tools, diagnostic criteria for outcome events, etc). For all biomarker outcomes from each included study, we will systematically extract detailed methodology including the specific assay technique (eg, enzyme-linked immunosorbent assay or chemiluminescence immunoassay), sampling time points, biomarker reference ranges, and units of measurement. Missing data will be retrieved from corresponding authors via email. Studies with irretrievable critical data will be excluded from quantitative synthesis. For duplicate publication of outcome data, we will retain only the most comprehensive or recent publication to avoid data overlap.

### Risk of Bias Assessment

Two investigators (CW and XW) will independently assess the methodological quality of included studies using the revised Cochrane risk of bias tool for randomized trials (RoB 2) [34]. The evaluation will cover 7 domains: random sequence generation, allocation concealment, blinding of participants and personnel, blinding of outcome assessment, incomplete outcome data, selective reporting, and other biases. Searching for and comparing preregistered trial protocols will be an integral part of identifying selective reporting. Each domain will be judged as “low risk,” “high risk,” or “unclear risk,” with discrepancies arbitrated by KW.

### Data Synthesis and Analysis

All meta-analyses will be performed using RevMan (version 5.4; Cochrane Collaboration). Mean differences and 95% CI will be used to calculate the effect size for continuous variables, and relative risk estimates with 95% CI will be used for dichotomous variables. When the units of continuous variables are not standardized, the standardized mean differences with 95% CI will be used. Heterogeneity will be assessed via *I*² statistic and *Q* test. If *I*²≤50% and *P*≥.1, indicating acceptable heterogeneity, a fixed-effect model will be used; if *I*²>50% or *P*<.1, suggesting substantial heterogeneity, a random-effects model will be selected [35]. Forest plots will be generated to visually present the meta-analysis results, with *P*<.05 considered statistically significant (2-tailed). Where feasible, independent analyses for POD and POCD will be performed to investigate the potential differential effects of electroacupuncture. For biomarker outcomes, quantitative synthesis will be attempted only for studies with comparable methodologies and sampling time points (eg, enzyme-linked immunosorbent assay results from samples taken 24 hours postoperatively). Where quantitative synthesis is precluded by clinical or methodological heterogeneity (eg, vast differences in timing and methods or too few studies per group), we will perform a systematic narrative synthesis and summarize key findings in structured evidence tables.

### Subgroup Analyses and Sensitivity Analyses

Given the anticipated clinical heterogeneity in electroacupuncture interventions, we will use the following strategies: (1) detailed description of electroacupuncture parameters (eg, acupoints, stimulation parameters, and timing) for all included studies; (2) prespecified subgroup analyses to explore the impact of key sources of heterogeneity, including acupoint selection (eg, scalp vs body acupoints and standardized vs individualized prescriptions), stimulation frequency (eg, 2 Hz vs >20 Hz), the number of electroacupuncture sessions (multiple sessions [≥2] vs single session), and electroacupuncture intervention timing (preoperative, intraoperative, postoperative, or multiphase perioperative intervention); and (3) if a sufficient number of studies are available (eg, ≥10 studies), meta-regression will be conducted to assess the contribution of continuous variables (eg, the total number of electroacupuncture sessions) to the observed heterogeneity. The results of these analyses will be integral to the interpretation of the pooled findings. Additionally, procedure-stratified and evaluation time–stratified subgroup analyses will also be conducted to reveal differential effect sizes contributed by surgical trauma intensity and outcome assessment time points. Sensitivity analyses will be performed by sequentially excluding each low-quality study with high overall risk of bias, aiming to evaluate the robustness of the findings and determine whether potential methodological bias exists in these studies.

### Evaluation of Publication Bias

When more than 10 studies are included for a specific outcome, we will assess publication bias through funnel plot asymmetry test and Egger linear regression test, with a statistical significance threshold of *P*<.05 indicating potential publication bias [36].

### Evidence Certainty Assessment

Two reviewers (CW and XW) will independently grade the evidence certainty for each critical outcome using the Grading of Recommendations Assessments, Developments and Evaluations (GRADE) approach [37], yielding classifications of “high,” “moderate,” “low,” or “very low” confidence. Disagreements will be resolved through discussion or third-party (KW) adjudication. The GRADE assessment will evaluate 5 domains: risk of bias, inconsistency, indirectness, imprecision, and other considerations. The assessment of “risk of bias” will be informed by the overall RoB 2 judgment. The certainty of evidence will be downgraded for risk of bias if a substantial proportion of the contributing studies are judged to be at high risk in key domains. The certainty of evidence will be downgraded for imprecision if the included studies have limited sample sizes that fail to meet the optimal information size criterion. If the included studies are found to have potential publication bias, the certainty of evidence will be downgraded within the “other considerations” domain.

### Patient and Public Involvement

Patients and the public will not be involved in the design, conduct, data analysis, interpretation of results, reporting, and dissemination plans of this study.

### Ethical Considerations

This study does not involve the direct participation of patients and the public, so ethics approval and informed consent are not applicable and required. Because the systematic review relies on previously published data, no original data will be collected.

### Validity, Reliability, and Rigor

The study will be rigorously conducted following the Cochrane Handbook for Systematic Reviews of Interventions [30] and will comply with the PRISMA 2020 guidelines [31] to guarantee transparency and comprehensive reporting.

### Amendments

The protocol for this systematic review will be amended when necessary, and the revisions will be documented in PROSPERO.

## Results

**Figure 1.**
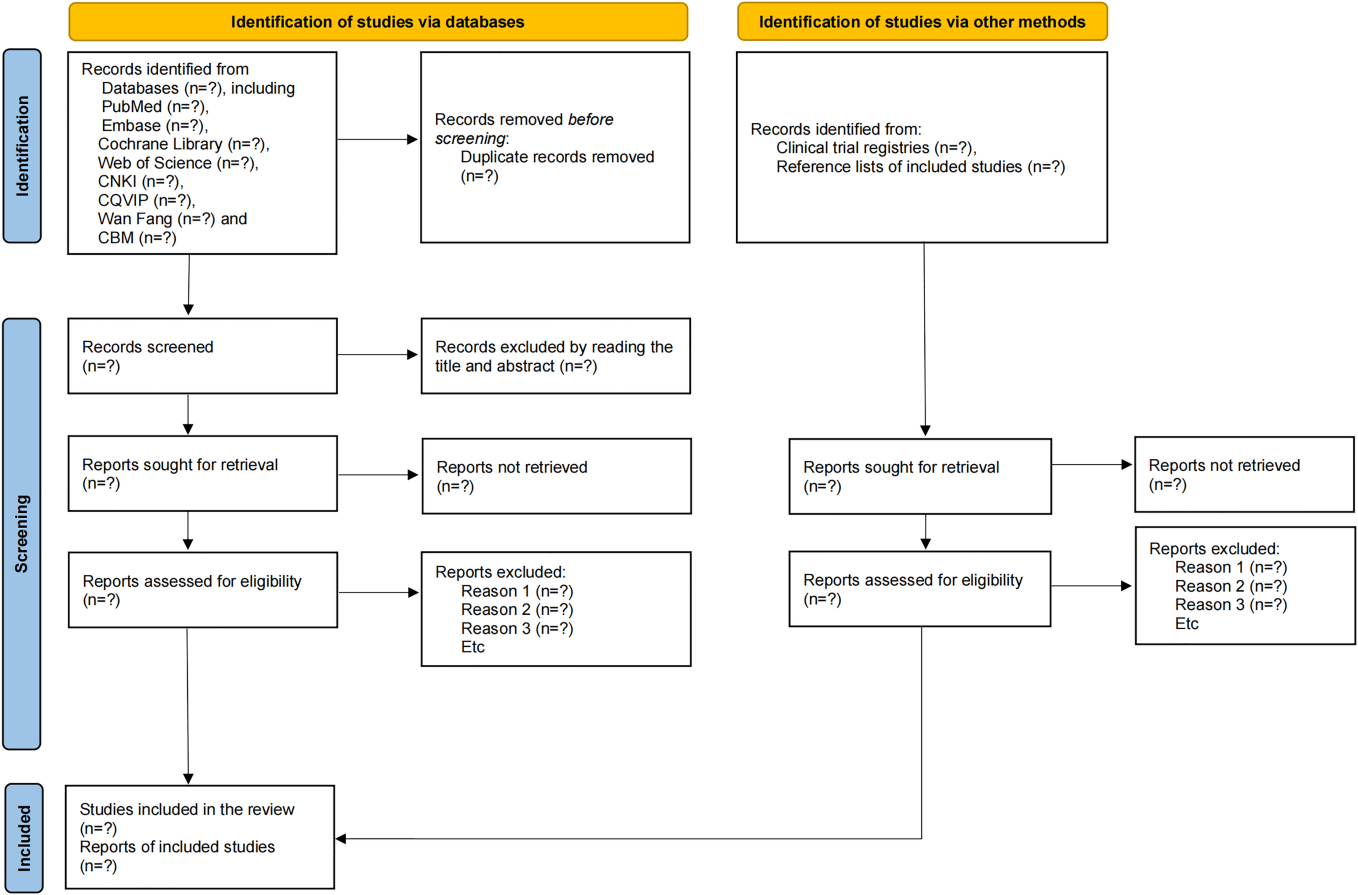
PRISMA (Preferred Reporting Items for Systematic Reviews and Meta-Analyses) flow diagram of study selection process. CBM: China Biomedical Literature Database; CNKI: China National Knowledge Infrastructure; CQVIP: Chongqing VIP Chinese Science and Technology Periodical Database.

The study selection process will be detailed using a PRISMA [31] flow diagram (Figure 1), which will document the number of records identified through database searching and the number of studies screened, assessed for eligibility, and ultimately included in the review, along with reasons for exclusions at each stage. Characteristics (design, populations, intervention protocols [eg, specific acupoints, waveform, frequency, intensity, duration per session, total number of sessions, and electroacupuncture intervention timing], comparisons, outcome measures, etc) of included RCTs will be tabulated. Methodological quality will be assessed by the RoB 2 tool and visualized graphically. All eligible studies will be included regardless of their risk of bias to avoid introducing selection bias. Data will be synthesized and analyzed using appropriate meta-analytic models where feasible, with forest plots for presentation (reporting relative risk, mean difference, or standardized mean difference with 95% CI). The results of subgroup analyses, sensitivity analyses, and evidence quality will be displayed in tables, while funnel plots will be used to evaluate publication bias. Should substantial heterogeneity preclude a meaningful meta-analysis for any outcome, the results will be synthesized narratively and presented in structured summary tables.

The findings of this study will be submitted for publication in a peer-reviewed journal. To keep the review up to date, we will conduct quarterly updates of all database searches (including gray literature sources) and incorporate any newly available evidence through a standardized living systematic review approach, with protocol revisions documented in PROSPERO.

The protocol was funded in March 2025. The literature search has been completed as of April 2025; an updated search will be conducted through December 31, 2025. Data extraction is projected to start on January 15, 2026, and end on January 31, 2026. Data analysis is planned to begin on February 1, 2026. Results are expected to be submitted for publication in March 2026.

## Discussion

### Clinical Dilemma of PND

Advanced age (≥60 years, particularly ≥70 years) and general anesthesia are established independent risk factors for PND [1,3]. Epidemiological data demonstrate a marked age-dependent increase in PND incidence, with patients aged 70 years and older experiencing twice the risk compared with those aged 60 to 69 years [38]. The confluence of population aging and rising surgical volumes has rendered PND a critical public health challenge, adversely affecting both short-term recovery trajectories and long-term quality of life, while compounding societal health care burdens [1,39]. To date, no effective treatment exists for PND, underscoring the critical importance of exploring a novel preventive strategy.

### Potential Impact of Electroacupuncture on PND

Accumulating evidence supports acupuncture’s neuroprotective potential, with studies reporting significant cognitive improvements across multiple disorders including Alzheimer disease [18], mild cognitive impairment [40], vascular dementia [20], vascular cognitive impairment [41], poststroke cognitive impairment [42], schizophrenia [43], and cognitive dysfunction following acute myocardial ischemia-reperfusion injury [17]. Clinical studies [12,13,16] have confirmed electroacupuncture’s potential to reduce PND incidence and improve cognitive recovery in older patients receiving general anesthesia. Electroacupuncture may exert neuroprotective effects through multifaceted mechanisms such as (1) attenuation of neuroinflammation and systemic inflammatory responses [18,44], potentially mediated via the vagal-adrenal axis activation [44]; (2) remodeling of synaptic structural and functional plasticity [18]; (3) upregulation of brain-derived neurotrophic factor related to neuronal survival and synaptic plasticity [45,46]; (4) modulation of gut microbiota homeostasis [19]; (5) inhibition of microglial activation [17,19]; (6) prevention of neuronal apoptosis [17]; (7) mitigation of oxidative stress [21]; and (8) enhancement of cerebral perfusion [20]. Neuroimaging evidence indicates that acupuncture can activate and enhance neural activity in cognition-related brain regions (eg, prefrontal cortex and hypothalamus) [18,40], while strengthening functional connectivity to modulate brain networks [47]. This suggests that the molecular effects of electroacupuncture ultimately translate into enhanced neural efficiency and network integration, providing a plausible substrate for observed cognitive benefits. Furthermore, acupuncture provides complementary perioperative benefits [48,49] such as preoperative anxiety relief, intraoperative anesthetic requirement reduction, attenuation of surgical stress responses, and postoperative complication decrease, collectively contributing to PND prevention [1]. In addition, electroacupuncture demonstrates unique integrative advantages by combining acupoint specificity, electrical stimulation, and needling effects. Its favorable safety profile, minimal invasiveness, and lower cost-effectiveness render it particularly suitable for perioperative application.

### Evidence Gaps in Existing Research

Current evidence remains fragmented and insufficient due to clinical heterogeneity, methodological heterogeneity, and limited sample sizes in existing studies [12-16,22], which consequently restricts the widespread perioperative application of electroacupuncture. While previous systematic reviews and meta-analyses [23-28] have primarily focused on transcutaneous electrical acupoint stimulation for PND, there remains an evidence gap regarding electroacupuncture’s potential for PND prevention in older patients (≥60 years) undergoing general anesthesia. Therefore, we propose this review to objectively evaluate the effectiveness and safety of perioperative electroacupuncture for PND prevention in this high-risk population, with the ultimate goal of providing updated evidence-based recommendations for clinical practice.

### Rationale for Outcome Selection

The incidence of PND will serve as the primary outcome, providing direct evidence on whether electroacupuncture reduces the risk of PND. The MMSE scores are selected as a secondary outcome due to its well-established sensitivity and specificity in assessing global cognitive function [32], thereby comprehensively capturing electroacupuncture’s potential benefits on postoperative cognitive recovery. Neuroinflammation represents one of the most critical pathological mechanisms underlying PND [50]. Specifically, the systemic inflammatory response syndrome triggered by surgical trauma and stress exacerbates central nervous system inflammation through multiple pathways: (1) increased blood-brain barrier permeability, (2) activation of microglia and astrocytes, and (3) subsequent release of proinflammatory cytokines (eg, IL-1β, IL-6, and TNF-α) into the central nervous system. These processes collectively contribute to synaptic dysfunction, neuronal injury or death, impaired hippocampal neurogenesis, and loss of synaptic plasticity, ultimately leading to PND. Neuronal damage is further reflected by elevated levels of brain injury biomarkers (NSE and S100β). These proteins enter systemic circulation via a compromised blood-brain barrier. Clinical studies have consistently demonstrated significantly increased serum concentrations of NSE and S100β in patients with PND following coronary artery bypass grafting [51], abdominal surgery [52], and orthopedic procedures [53]. Therefore, this study selects serum levels of IL-1β, IL-6, TNF-α, NSE, and S100β as secondary outcome measures to evaluate whether electroacupuncture exerts neuroprotective effects by mitigating peripheral inflammatory responses and reducing brain injury severity. These outcomes will help establish an “intervention-effectiveness-mechanism” framework, providing critical insights into electroacupuncture’s perioperative neuroprotection.

### Strengths, Anticipated Results, and Clinical Implications

This study will address a critical evidence gap by specifically investigating electroacupuncture’s effectiveness for PND prevention in older patients (≥60 years) undergoing elective general anesthesia—a previously unexamined yet clinically significant population. Through prespecified subgroup and sensitivity analyses, we will determine how key covariates influence intervention effects and verify the robustness of results. Furthermore, the evaluation of methodological rigor in existing RCTs will identify crucial knowledge gaps to inform the design of future high-quality clinical trials.

Should meta-analysis confirm electroacupuncture’s effectiveness and safety for PND prevention among older patients undergoing surgery, the GRADE-assessed evidence from this review will (1) qualify electroacupuncture as a candidate intervention for inclusion in clinical guidelines, (2) offer a novel nonpharmacological strategy to optimize perioperative care protocols for older adults, and (3) potentially mitigate the cascade of adverse outcomes associated with PND through enhanced cognitive recovery in high-risk populations. Such confirmation would represent a significant advancement in clinical practice, particularly for anesthesia and surgical teams managing older patients.

### Limitations

This study has several inherent limitations: First, clinical heterogeneity is anticipated due to variations across studies in both demographic factors (anesthesia regimens and surgical types) and electroacupuncture intervention protocols (eg, acupuncture prescription, intervention timing, sessions, and stimulation parameters), which may preclude pooled quantitative analysis for certain outcomes. Second, methodological limitations in the included studies may compromise the strength of the evidence. Third, the generalizability of findings may be restricted to older patients undergoing elective surgery under general anesthesia, excluding those requiring emergency procedures. Future research should (1) conduct multicenter RCTs with standardized electroacupuncture protocols (including optimal acupoint selection, waveform, frequency, and intervention timing) and improved methodology (eg, standardized outcome assessment protocols), (2) elucidate electroacupuncture’s mechanisms of action by correlating biomarkers with multimodal neuroimaging data, and (3) develop risk stratification tools to identify patients most likely to benefit from electroacupuncture intervention.

### Conclusions

This systematic review will provide a comprehensive, GRADE-assessed evidence synthesis specifically focused on the efficacy and safety of electroacupuncture for PND prevention in older patients undergoing general anesthesia. The findings are expected to either support electroacupuncture’s integration into ERAS protocols if effectiveness is demonstrated or identify critical knowledge gaps requiring further RCTs if evidence remains inconclusive.

## Supplementary material

10.2196/84010 Multimedia Appendix 1 Search strategies.

10.2196/84010 Checklist 1 PRISMA-P checklist.

## Abbreviations

ERAS: Enhanced Recovery After Surgery
GRADE: Grading of Recommendations Assessments, Developments and Evaluations
IL-1β: interleukin-1β
IL-6: interleukin-6
MeSH: Medical Subject Headings
MMSE: Mini-Mental State Examination
MoCA: Montreal Cognitive Assessment
NSE: neuron-specific enolase
PND: perioperative neurocognitive disorder
POCD: postoperative cognitive dysfunction
POD: postoperative delirium
PRISMA: Preferred Reporting Items for Systematic Reviews and Meta-Analyses
PRISMA-P: Preferred Reporting Items for Systematic Reviews and Meta-Analyses Protocols
PROSPERO: International Prospective Register of Systematic Reviews
RCT: randomized controlled trial
RoB 2: revised Cochrane Risk of Bias tool for randomized trials
S100β: S100 calcium-binding protein β
TNF-α: tumor necrosis factor-α
