# Supplementary material for: Electroacupuncture for the Prevention of Perioperative Neurocognitive Disorder in Older Patients Undergoing General Anesthesia: Protocol for a Systematic Review and Meta-Analysis
Source: JMIR Res Protoc. 2025 Dec 30;14:e84010. doi: 10.2196/84010 (PMC12752915; doi:10.2196/84010)
Supplement: Multimedia Appendix 1 [file resprot-v14-e84010-s001.docx]

**Table S1.** The detailed search strategy.

| **Electronic databases** | **Search** | **Search strategy** | **Results** |
| --- | --- | --- | --- |
| **PubMed** | #1 | (cognition) OR (neurocognitive disorders) OR (cognitive dysfunction) OR (cognition disorders) OR (delirium) [MeSH Terms] | 27 |
|  | #2 | (cognitive disorders) OR (cognitive impairment) OR (cognitive decline) OR (cognition dysfunction) OR (cognition impairment) OR (cognition decline) OR (cognit*) [Title/Abstract] |  |
|  | #3 | #1 OR #2 |  |
|  | #4 | (postoperative period) OR (anesthesia recovery period) OR (postop*) OR (postoperative*) OR (postoperative) OR (postsurgical*) OR (post anesthesia) OR (after surgery) OR (post-surgery) [Title/Abstract] |  |
|  | #5 | #3 AND #4 |  |
|  | #6 | (postoperative cognitive complications) [MeSH Terms] |  |
|  | #7 | (delayed neurocognitive recovery) OR (perioperative neurocognitive disorder) OR (perioperative cognitive disorder) OR (POCD) OR (POD) OR (PND) [Title/Abstract] |  |
|  | #8 | #5 OR #6 OR #7 |  |
|  | #9 | (acupuncture) OR (electroacupuncture) OR (acupuncture therapy) [MeSH Terms] |  |
|  | #10 | (aged) [MeSH Terms] |  |
|  | #11 | (elderly) OR (older) OR (geriatric) [Title/Abstract] |  |
|  | #12 | #10 OR #11 |  |
|  | #13 | (randomized controlled trial) OR (controlled clinical trial) OR (random*) OR (randomization) OR (randomly) [Title/Abstract] |  |
|  | #14 | #8 AND #9 AND #12 AND #13 |  |
| **Cochrane** | #1 | ((neurocognitive disorders) OR (cognitive dysfunction) OR (cognition disorders) OR (cognitive disorders) OR (cognitive impairment) OR (cognitive decline) OR (cognition dysfunction) OR (cognition impairment) OR (cognition decline) OR (cognit*) OR (delirium)):ti,ab,kw | 47 |
|  | #2 | MeSH descriptor: [Cognition] explode all trees |  |
| **Electronic databases**  **Supplementary Table S1.** (Continued) | **Search** | **Search strategy** | **Results** |
| **Cochrane** | #3 | MeSH descriptor: [Neurocognitive Disorders] explode all trees | 47 |
|  | #4 | MeSH descriptor: [Cognitive Dysfunction] explode all trees |  |
|  | #5 | MeSH descriptor: [Cognition Disorders] explode all trees |  |
|  | #6 | MeSH descriptor: [Delirium] explode all trees |  |
|  | #7 | #1 OR #2 OR #3 OR #4 OR #5 OR #6 |  |
|  | #8 | ((postoperative period) OR (anesthesia recovery period) OR (postop*) OR (postoperative*) OR (postoperative) OR (postsurgical*) OR (post anesthesia) OR (after surgery) OR (post-surgery)):ti,ab,kw |  |
|  | #9 | MeSH descriptor: [Postoperative Period] explode all trees |  |
|  | #10 | MeSH descriptor: [Anesthesia Recovery Period] explode all trees |  |
|  | #11 | #8 OR #9 OR #10 |  |
|  | #12 | #7 AND #11 |  |
|  | #13 | ((postoperative cognitive complications) OR (delayed neurocognitive recovery) OR (perioperative neurocognitive disorder) OR (perioperative cognitive disorder) OR (POCD)):ti,ab,kw |  |
|  | #14 | MeSH descriptor: [Postoperative Cognitive Complications] explode all trees |  |
|  | #15 | #12 OR #13 OR #14 |  |
|  | #16 | ((acupuncture) OR (electroacupuncture) OR (EA)):ti,ab,kw |  |
|  | #17 | MeSH descriptor: [Acupuncture] explode all trees |  |
|  | #18 | MeSH descriptor: [Acupuncture Therapy] explode all trees |  |
|  | #19 | MeSH descriptor: [Electroacupuncture] explode all trees |  |
|  | #20 | #16 OR #17 OR #18 OR #19 |  |
|  | #21 | MeSH descriptor: [aged] explode all trees |  |
|  | #22 | ((elderly) OR (older) OR (geriatric)):ti,ab,kw |  |
|  | #23 | #21 OR #22 |  |
|  | #24 | ((randomized controlled trial) OR (controlled clinical trial) OR (random*) OR (randomization) OR (randomly)):ti,ab,kw |  |
| **Electronic databases**  **Supplementary Table S1.** (Continued) | **Search** | **Search strategy** | **Results** |
| **Cochrane** | #25 | MeSH descriptor: [Randomized Controlled Trial] explode all trees | 47 |
|  | #26 | MeSH descriptor: [Controlled Clinical Trial] explode all trees |  |
|  | #27 | #24 OR #25 OR #26 |  |
|  | #28 | #15 AND #20 AND #23 AND #27 |  |
| **Web of science** | #1 | TS=(“neurocognitive disorders” OR “cognitive dysfunction” OR “cognition disorders” OR “cognitive disorders” OR “cognitive impairment” OR “cognitive decline” OR “cognition dysfunction” OR “cognition impairment” OR “cognition decline” OR “cognit*” OR “delirium”) | 39 |
|  | #2 | TS=(“postoperative period” OR “anesthesia recovery period” OR “postop*” OR “postoperative*” OR “postoperative” OR “postsurgical*” OR “post anesthesia” OR “after surgery” OR “post-surgery”) |  |
|  | #3 | #1 AND #2 |  |
|  | #4 | TS=(“postoperative cognitive complications” OR “delayed neurocognitive recovery” OR “perioperative neurocognitive disorder” OR “perioperative cognitive disorder” OR “POCD” OR “POD” OR “PND”) |  |
|  | #5 | #3 OR #4 |  |
|  | #6 | TS=(“acupuncture” OR “electroacupuncture” OR “EA”) |  |
|  | #7 | TS=(“aged” OR “elderly” OR “older” OR “geriatric”) |  |
|  | #8 | TS=(“randomized controlled trial” OR “controlled clinical trial” OR “random*” OR “randomization” OR “randomly”) |  |
|  | #9 | #5 AND #6 AND #7 AND #8 |  |
| **Embase** | #1 | 'cognition'/exp OR cognition | 138 |
|  | #2 | 'disorders of higher cerebral function'/exp OR 'disorders of higher cerebral function' OR (('disorders'/exp OR disorders) AND of AND higher AND cerebral AND ('function'/exp OR function)) |  |
|  | #3 | 'cognitive defect'/exp OR 'cognitive defect' OR (cognitive AND defect) |  |
|  | #4 | 'delirium'/exp OR delirium |  |
|  | #5 | 'neurocognitive disorders'/exp OR 'neurocognitive disorders' OR (neurocognitive AND ('disorders'/exp OR disorders)) |  |
|  | #6 | 'cognitive dysfunction'/exp OR 'cognitive dysfunction' OR (cognitive AND dysfunction) |  |
|  | #7 | 'cognition disorders'/exp OR 'cognition disorders' OR (('cognition'/exp OR cognition) AND ('disorders'/exp OR |  |
| **Electronic databases**  **Supplementary Table S1.** (Continued) | **Search** | **Search strategy** | **Results** |
| **Embase** | #7 | disorders)) | 138 |
|  | #8 | 'cognitive disorders'/exp OR 'cognitive disorders' OR (cognitive AND ('disorders'/exp OR disorders)) |  |
|  | #9 | 'cognitive impairment'/exp OR 'cognitive impairment' OR (cognitive AND ('impairment'/exp OR impairment)) |  |
|  | #10 | 'cognitive decline'/exp OR 'cognitive decline' OR (cognitive AND ('decline'/exp OR decline)) |  |
|  | #11 | 'cognition dysfunction' OR (('cognition'/exp OR cognition) AND dysfunction) |  |
|  | #12 | 'cognition impairment' OR (('cognition'/exp OR cognition) AND ('impairment'/exp OR impairment)) |  |
|  | #13 | 'cognition decline' OR (('cognition'/exp OR cognition) AND ('decline'/exp OR decline)) |  |
|  | #14 | cognit* |  |
|  | #15 | #1 OR #2 OR #3 OR #4 OR #5 OR #6 OR #7 OR #8 OR #9 OR #10 OR #11 OR #12 OR #13 OR #14 |  |
|  | #16 | 'postoperative period'/exp OR 'postoperative period' OR (postoperative AND period) |  |
|  | #17 | 'anesthetic recovery'/exp OR 'anesthetic recovery' OR (('anesthetic'/exp OR anesthetic) AND ('recovery'/exp OR recovery)) |  |
|  | #18 | 'anesthesia recovery period'/exp OR 'anesthesia recovery period' OR (('anesthesia'/exp OR anesthesia) AND ('recovery'/exp OR recovery) AND period) |  |
|  | #19 | #16 OR #17 OR #18 |  |
|  | #20 | #15 AND #19 |  |
|  | #21 | 'postoperative cognitive dysfunction'/exp OR 'postoperative cognitive dysfunction' OR (postoperative AND cognitive AND dysfunction) |  |
|  | #22 | 'postoperative cognitive complications'/exp OR 'postoperative cognitive complications' OR (postoperative AND cognitive AND ('complications'/exp OR complications)) |  |
|  | #23 | 'delayed neurocognitive recovery'/exp OR 'delayed neurocognitive recovery' OR (delayed AND neurocognitive AND ('recovery'/exp OR recovery)) |  |
|  | #24 | 'perioperative neurocognitive disorder'/exp OR 'perioperative neurocognitive disorder' OR (perioperative AND neurocognitive AND ('disorder'/exp OR disorder)) |  |
| **Electronic databases**  **Supplementary Table S1.** (Continued) | **Search** | **Search strategy** | **Results** |
| **Embase** | #25 | 'perioperative cognitive disorder' OR (perioperative AND cognitive AND ('disorder'/exp OR disorder)) | 138 |
|  | #26 | pocd |  |
|  | #27 | #21 OR #22 OR #23 OR #24 OR #25 OR #26 |  |
|  | #28 | #20 OR #27 |  |
|  | #29 | 'acupuncture'/exp OR acupuncture |  |
|  | #30 | 'electroacupuncture'/exp OR electroacupuncture |  |
|  | #31 | ea |  |
|  | #32 | #29 OR #30 OR #31 |  |
|  | #33 | 'randomized controlled trial'/exp OR 'randomized controlled trial' OR (randomized AND controlled AND ('trial'/exp OR trial)) |  |
|  | #34 | 'controlled clinical trial'/exp OR 'controlled clinical trial' OR (controlled AND ('clinical'/exp OR clinical) AND ('trial'/exp OR trial)) |  |
|  | #35 | random* |  |
|  | #36 | 'randomization'/exp OR randomization |  |
|  | #37 | randomly |  |
|  | #38 | #33 OR #34 OR #35 OR #36 OR #37 |  |
|  | #39 | 'aged'/exp OR 'aged' |  |
|  | #40 | 'elderly'/exp OR elderly |  |
|  | #41 | 'older adult'/exp OR 'older adult' OR (older AND ('adult'/exp OR adult)) |  |
|  | #42 | 'geriatric'/exp OR geriatric |  |
|  | #43 | #39 OR #40 OR #41 OR #42 |  |
|  | #44 | #28 AND #32 AND #38 AND #43 |  |
| **CBM** | #1 | 针刺 OR 针刺疗法OR 电针 OR 电针疗法 OR “EA”（使用快速检索） | 139 |
|  | #2 | 认知 OR 认知障碍 OR 认知功能障碍 OR 神经认知障碍 OR 认知功能损害 OR 认知能力下降 OR 认 |  |
| **Electronic databases**  **Supplementary Table S1.** (Continued) | **Search** | **Search strategy** | **Results** |
| **CBM** | #2 | 知下降 OR 术后认知并发症 OR 认知恢复延迟 OR 谵妄 OR 精神错乱 OR 躁动 OR “POCD” OR “POD” OR “PND”（使用快速检索） | 139 |
|  | #3 | 围手术期 OR 麻醉恢复期 OR 术后（使用快速检索） |  |
|  | #4 | 随机对照试验 OR 临床对照试验 OR 临床试验 OR 多中心研究 OR 随机对照 OR 随机 OR 疗效观察（使用快速检索） |  |
|  | #5 | 老年 OR 老人 OR 高龄 OR 60岁（使用快速检索） |  |
|  | #6 | #1 AND #2 AND #3 AND #4 AND #5 |  |
| **CNKI** | #1 | (SU=(“针刺”+“针刺疗法”+“电针”+“电针疗法”+“EA”) OR TKA=(“针刺”+“针刺疗法”+“电针”+“电针疗法”+“EA”)) AND (SU=(“认知”+“认知障碍”+“认知功能障碍”+“神经认知障碍”+“认知损害”+“认知下降”+“认知减退”+“术后认知并发症”+“记忆障碍”+“认知恢复延迟”+“谵妄”+“精神错乱”+“躁动”+“PND”+“POD”+“POCD”) OR TKA=(“认知”+“认知障碍”+“认知功能障碍”+“神经认知障碍”+“认知损害”+“认知下降”+“认知减退”+“术后认知并发症”+“记忆障碍”+“认知恢复延迟”+“谵妄”+“精神错乱”+“躁动”+“PND”+“POD”+“POCD”)) AND (TKA=(“术后”+“围手术期”+“麻醉恢复期”)) AND (TKA=(“随机对照试验”+“临床对照试验”+“临床试验”+“随机对照”+“随机”+“多中心”+“疗效观察”))AND (TKA=(“老年”+“老人”+“高龄”+“60岁”)) | 412 |
| **Wan Fang** | #1 | (题名或关键词:(“针刺” OR “针刺疗法” OR “电针” OR “电针疗法” OR “EA”) OR 摘要:(“针刺” OR “针刺疗法” OR “电针” OR “电针疗法” OR “EA”)) AND (题名或关键词:(“认知障碍” OR “认知功能障碍” OR “神经认知障碍” OR “认知损害” OR “认知下降” OR “认知减退” OR “术后认知并发症” OR “认知恢复延迟” OR “谵妄” OR “POCD” OR “POD” OR “PND”) OR 摘要:(“认知障碍” OR “认知功能障碍” OR “神经认知障碍” OR “认知损害” OR “认知下降” OR “认知减退” OR “术后认知并发症” OR “认知恢复延迟” OR “谵妄” OR “POCD” OR “POD” OR “PND”)) AND (题名或关键词:(“术后” OR “围手术期” OR “麻醉恢复期”) AND (全部:(“临床试验” OR “随机对照” OR “随机” OR “临床研究” OR “疗效观察”)) AND (题名或关键词:(“老年” OR “老人” OR “高龄” OR “60岁”)) | 1901 |
| **Electronic databases** | **Search** | **Search strategy** | **Results** |
| **VIP** | #1 | (M=(针刺 OR 针刺疗法 OR 电针 OR 电针疗法 OR “EA”) OR R=(针刺 OR 针刺疗法 OR 电针 OR 电针疗法 OR “EA”)) AND (M=(认知 OR 认知障碍 OR 认知功能障碍 OR 神经认知障碍 OR 认知损害 OR 认知下降 OR 认知减退 OR 术后认知并发症 OR 认知恢复延迟 OR 谵妄 OR “POCD” OR “POD” OR “PND”) OR R=(认知 OR 认知障碍 OR 认知功能障碍 OR 神经认知障碍 OR 认知损害 OR 认知下降 OR 认知减退 OR 术后认知并发症 OR 认知恢复延迟 OR 谵妄 OR “POCD” OR “POD” OR “PND”)) AND (M=(术后 OR 围手术期 OR 麻醉恢复期)) AND (U=(随机对照试验 OR 临床对照试验 OR 临床试验 OR 随机对照 OR 随机 OR 多中心 OR 疗效观察)) AND (M=(老年 OR 老人 OR 高龄 OR “60岁”)) | 73 |
| [**http://www.chictr.org.cn/**](http://www.chictr.org.cn/) | #1 | 注册题目= (认知 OR 认知损害 OR 认知下降 OR 认知减退 OR 记忆障碍 OR 认知恢复延迟 OR 术后认知障碍 OR 术后认知功能障碍 OR 术后神经认知障碍 OR 术后认知并发症 OR 术后谵妄)  手动选择= (探索性研究/预试验/0 OR 随机平行对照/Parallel OR 干预性研究/Interventional study) | 55 |
| **ClinicalTrials.gov** | #1 | Condition/disease= (“neurocognitive disorders” OR “cognitive dysfunction” OR “cognition disorders” OR “cognitive disorders” OR “cognitive impairment” OR “cognitive decline” OR “cognition dysfunction” OR “cognition impairment” OR “cognition decline” OR “cognit*” OR “delirium” OR “postoperative cognitive complications” OR “delayed neurocognitive recovery” OR “postoperative cognitive dysfunction” OR “postoperative delirium” OR “perioperative neurocognitive disorder” OR “perioperative cognitive disorder” OR “POCD” OR “POD” OR “PND”) | 10 |
|  |  | Intervention/treatment= (“acupuncture” OR “electroacupuncture” OR “EA”)  Other terms= (“elderly” OR “older” OR “geriatric”)  Manually enter range= From “60” Years Old To “80” Years Old |  |
| [**http://itmctr.ccebtcm.org.cn/**](http://itmctr.ccebtcm.org.cn/) | #1 | Disease= (“neurocognitive disorders” OR “cognitive dysfunction” OR “cognition disorders” OR “cognitive disorders” OR “cognitive impairment” OR “cognitive decline” OR “cognition dysfunction” OR “cognition impairment” OR “cognition decline” OR “cognit*” OR “delirium” OR “postoperative cognitive complications” OR “delayed neurocognitive recovery” OR “postoperative cognitive dysfunction” OR “postoperative delirium” OR “perioperative neurocognitive disorder” OR “perioperative cognitive disorder” OR “POCD” OR “POD” OR “PND”)  Intervention= (“acupuncture” OR “electroacupuncture” OR “EA” | 12 |

**Supplementary Table S1.** (Continued)

**Supplementary Table S2.** (Continued)
